# Exploring the Sensitivity of Peripheral ADA Levels Measurement in Establishing Psychosis Susceptibility

**DOI:** 10.1007/s12031-025-02362-3

**Published:** 2025-05-29

**Authors:** S. Cilem Kizilpinar, F. Bahar Atak-Akkus, Ozlem Dogan, Burcin Colak, M. Cigdem Aydemir

**Affiliations:** 1 Department of Psychiatry, Adana City Research and Training Hospital, Ministry of Health, Adana, Turkey; 2 Department of Psychiatry, Bilkent City Research and Training Hospital, Ministry of Health, Ankara, Turkey; 3 Department of Biochemistry, Faculty of Medicine, Ankara University, Ankara, Turkey; 4 Department of Psychiatry, Faculty of Medicine, Ankara University, Ankara, Turkey

**Keywords:** Adenosine deaminase, Binary logistic regression, Clinical high risk, Psychosis, Schizophrenia

## Abstract

Studies on the relationship between the adenosinergic system and schizophrenia have been released, but none has explored the relationship between adenosine deaminase and psychosis risk. Our primary objective is to investigate the sensitivity and specificity of peripheral adenosine deaminase enzyme levels regarding susceptibility to psychosis. In this cross-sectional case–control study, the serum levels of adenosine deaminase were compared among patients with schizophrenia, first-degree relatives of schizophrenia patients, and healthy controls. The patient and relative groups were classified as high-risk groups and healthy controls as low-risk groups. A binary logistic regression analysis was conducted to determine whether serum ADA levels can distinguish the low-risk group from the high-risk group. Healthy controls had higher serum ADA levels than the patient and relative groups (*p* = 0.019; *p* = 0.027). There was no statistically significant difference between patients and relatives (*p* = 0.998). Binary logistic regression analysis showed that serum ADA levels were 62.2% accurate in predicting psychosis risk, with a sensitivity of 82%. The results showed that serum ADA levels were significantly different between individuals at relatively low genetic risk (healthy controls) for schizophrenia and those at relatively high genetic risk (patients and relatives). According to the risk model based on serum ADA level, measuring serum ADA level may help distinguish genetically high-risk individuals from genetically low-risk individuals.

## Introduction

Schizophrenia is a complex clinical syndrome that impairs cognition, emotion, perception, speech, drive, and behavior (Labrie and Roder 2010; Tandon et al. 2013). Despite extensive research and treatment advances, schizophrenia still affects more than 1% of the general population and causes a significant burden on the patients and society (Howes and Murray 2014). Multiple hypotheses regarding the etiology of schizophrenia have been put forward, with the stress-diathesis model and gene-environment interactions emerging as particular (Carlsson 1988; Javitt 2010; Labrie and Roder 2010; van Os et al. 2010; Ciruela et al. 2015; Bernardo et al. 2017). Inflammatory pathways may also be associated with the pathophysiology of schizophrenia (Leza et al. 2015). A growing body of research indicates the importance of two-way interaction between the neural and immune systems for schizophrenia (Lara et al. 2006). In recent years, adenosinergic and purinergic systems, based on their neuromodulatory activity, became a focus as a potential candidate mechanism in schizophrenia (Ciruela et al. 2015; Leza et al. 2015).

Adenosine is a purine nucleoside that regulates neurotransmission in the brain. It has been suggested that the purinergic system regulates neurotransmission by affecting the dopaminergic, glutamatergic, noradrenergic, serotoninergic, and endocannabinoid systems and has neuroimmunological effects (Pasquini et al. 2022). A critical enzyme in this purinergic system is adenosine deaminase (ADA), a protein involved in the conversion of adenosine to inosine (Lara et al. 2006). It has been reported that ADA activity may have an effect on the clinical course of different medical and neuropsychiatric diseases, such as major depression, autism, and liver diseases (Rogers et al. 2001). In a study, ADA levels were higher in drug-naive, recent-onset schizophrenic patients than controls, and it was suggested that higher ADA levels lead to a hypoadenosynergic state by lowering adenosine levels (Sasidharan et al. 2017). A study of the activity of ADA in patients receiving antipsychotic therapy found a negative correlation between antipsychotic use and ADA activity. Furthermore, ADA and 5′ nucleotidase levels were correlated with antipsychotic treatment, particularly clozapine use (Brunstein et al. 2007). These results indicate that adenosine can be an important neuromodulator in the etiology of schizophrenia and is affected by treatment with antipsychotics. Adenosine likely plays an important role not only in the neurochemical hypothesis related to schizophrenia but also in the neurodevelopmental and neuroimmunological hypotheses. It has been suggested that neurotoxicity resulting from the dysfunction of adenosine A1R receptors during early brain development may increase the susceptibility to schizophrenia (Shenton et al. 2001; Lara et al. 2006). According to the neuroimmunological hypothesis, dysfunction of the adenosine metabolism may cause the imbalance of pro-inflammatory and anti-inflammatory cytokines that are critical for normal brain development, and this impairment may play a role in the development of schizophrenia (Haskó et al. 2005).

In recent years, as a means of limiting disability and reducing the extent of illness, preventative treatments have gained prominence in the diagnosis and treatment of schizophrenia. The first step of preventive interventions is to identify people at risk for the development of schizophrenia. Identifying the clinically high-risk (CHR) individuals with sensitive and specific methods is important to the efficiency and cost-effectiveness of preventive interventions. The recognition of the CHR condition for psychosis has resulted in a significant increase in literature on the subject and led to studies regarding the development of prognostic prediction models. Many risk groups for psychosis have currently been identified (Yung et al. 2004; Carpenter and van Os 2011; Vos et al. 2017). One common way to identify CHR among people is by checking for positive family histories of schizophrenia (Gottesman 1994). The biological, physical, and neuropsychiatric markers of psychosis risk among relatives of schizophrenia patients who are not affected by psychotic disorders help understand the etiology of schizophrenia. It has been suggested that familial vulnerability indicators might help comprehend the diseases’ heritable and/or familial components (Türközer et al. 2021). Curiosity about the etiology of schizophrenia and the need to individualize early intervention or preventive treatment for psychosis have promoted the investigation of predictive prediction models (Salazar et al. 2021).

Adenosine is a promising candidate for understanding the etiology of schizophrenia, developing treatment targets, and being a possible indicator for the diagnosis of psychosis. To our knowledge, there are some publications that investigate ADA level as an indicator of schizophrenia. However, no publications have investigated ADA level measurement as useful for the prediction of psychosis risk. Therefore, the purpose of the present study was (1) to evaluate the sensitivity and specificity of peripheral ADA levels in predicting the high risk of schizophrenia and (2) to explore the association between peripheral ADA levels and the clinical variables in the patient group.

## Materials and Methods

### Participants

Patients diagnosed with schizophrenia (PG, *n* = 29) based on the Diagnostic and Statistical Manual of Mental Disorders (DSM-5), first-degree relatives of these patients (RG, *n* = 25), and healthy controls without a family or personal history of autism spectrum disorders or psychosis (HCG, *n* = 34) were included in this cross-sectional case-control study. Patients who were in remission and had regular drug treatment for at least 3 months were admitted. All participant groups’ inclusion criteria consisted of being between the ages of 18 and 65, not having a history of substance or alcohol misuse, and not having a medical condition. Before drawing blood, participants were asked to refrain from fasting or smoking for at least 8 h because these actions might change adenosine levels.

### Assessment Procedures

Information on demographic variables such as age, gender, education levels, marital status, smoking and alcohol consumption, and substance use and data on various clinical characteristics such as the age of onset of first symptoms, the total duration of disease and medications (years), were obtained from all participants with sociodemographic variables form.

### Biochemical Measurements

Measurement of serum ADA activity was based on assessing the formation of ammonia produced in a Berthelot reaction. Ammonia is generated when ADA reacts with adenosine (substrate). In the final chemical reaction, indophenol’s formation of a blue color was measured with a spectrophotometer at 628 nm. ADA enzyme activity is expressed as IU/L. Absorbance readings for all methods were performed on the Shimadzu UVmini-1240 device (UV-VIS spectrophotometer, Shimadzu Corporation, Kyoto, Japan). Serum ADA activities were studied at the Medical Biochemistry Laboratory. All chemicals were purchased from Sigma Chemical (St. Louis, MO).

### Clinical Measurements

The Positive and Negative Syndrome Scale (PANSS): This scale provides data about the severity of the positive and negative symptoms and general psychopathology of patients with psychosis (Kay et al. 1987).

Clinical Global Impression Scale (CGI): The CGI was developed to assess the patient’s global functioning and symptom severity (Busner and Targum 2007). The 7-point Likert scale includes two parts, but only the CGI-Severity score was used in the study.

### Statistical Analysis

Initially, normality tests for continuous variables were evaluated with the Shapiro-Wilk test. Correlations were analyzed with Pearson or Spearman correlation tests according to whether the normality assumptions were met or not. An independent sample *t*-test and the Mann-Whitney *U* test were conducted to compare the two groups appropriately. One-way analysis of variance (ANOVA) was applied in multiple group comparisons. The Kruskal-Wallis test was used for multiple-group comparisons, and comparisons for categorical variables between groups were made using the Pearson Chi-Square Test. A binary logistic regression analysis with the enter method was conducted to analyze whether serum ADA levels can distinguish the healthy control group from the patient and the patient’s relative group. The patient and relative groups were classified as high-risk groups and healthy controls were classified as low-risk groups. Statistical analyses were performed with SPSS 26.0 (SPSS Inc, Chicago, IL, USA) package program, and the level of significance was accepted as *p* < 0.05.

## Results

### Sociodemographic and Clinical Features

A total of 90 participants have participated in three groups (PG=29, RG=27, HG=34). The detailed sociodemographic data and clinical characteristics of the participants are given in Table 1. A significant difference was found between the groups regarding gender (X^2^ = 6.195, p = 0.045) and smoking history (X^2^ = 7.106, p = 0.029). There was no statistically significant difference between the three groups in terms of age (H(2) = 5.07, p = 0.79) and education years (H(2) = 6.96, p = 0.79). 44.83% of the patients (3 or less, according to CGI) were determined as a mild group. 55.17% of patients (4 and above, according to CGI) were determined as a severe group. 13.8% of the patients used both typical and atypical antipsychotics (AP), and 86.2% used only atypical AP. There was no patient in our study who received only typical AP. 51.7% of the patients used only oral AP, 20.7% used long-acting injectable (LAI) antipsychotics, and 27.6% of the patients used both oral AP and LAI AP. The RG comprised 48.15% siblings, 37.0% parents, and 14.81% children. Since the sibling group was reported to have the most genetic similarity in previous studies, the RG was split into two, as siblings and others.

**Table 1:** Sociodemographic and clinical characteristics of the groups

Features	Patients (*n* = 29)	Relatives (*n* = 27)	Controls (*n* = 34)	Statistics	*p*-value
Age (year)	42.00 ± 11.30	47.44 ± 15.36	39.59 ± 12.96	*H* (2) = 5.07	*p*^K^ = 0.79
Gender (female)	11_a_* (38%)	19 _b_ (70%)	20 _a,b_ (58%)	*χ*2 = 6.195	*p* = 0,045
Education duration (year)	11.86 ± 0.56	10.96 ± 0.75	13.42 ± 0.69	*H* (2) = 6.96	*p*^K^ = 0.79
Marital status (married)	5_a_* (17.2%)	18_b_ (66.7%)	24_b_ (70,5%)	*χ*2 = 23.764	*p* = 0.009
ADA level (UI/L)	20.50 ± 9.71	20.71 ± 12.97	28.81 ± 12.71	*F(2,87)* = *5.022*	*p* = 0.009
Age of disease onset	23.79 ± 6.72				
Total duration of disorder (year)	18.21 ± 11.45				
Type of antipsychotic treatment n/(%)	Only typical AP, 0 Only atypical AP, 25 (86.21%) Typical and atypical AP combination, 4 (13.79%)				
PANSS (Positive)	12.24 ± 5.06				
PANSS (Negative)	16.34 ± 6.84				
PANSS (General)	27.21 ± 7.95				
PANSS *(*Total)	55.69 ± 16.09				
CGI (mild and moderately ill n, %)	13 (44.83%)				

Values are presented as mean ± standard deviation, number (%)

ADA adenosine deaminase, PANSS The Positive and Negative Syndrome Scale, CGI Clinical Global Impression Scale, AP Antipsychotic

*Each subscript letter denotes a subset of gender/marital status categories whose column proportions do not differ significantly from each other at the 0.5 level. If a category does not express a statistically significant difference between the two groups, it is denoted by the same letter. Categories that differ significantly are indicated by different letters

### Adenosine Deaminase

There was no significant difference between the genders regarding ADA level (t = −1.762, p = 0.082). Similarly, there was no difference between the groups in terms of ADA level when all participants were divided into two groups: smokers, non-smokers, and those who quit for at least 1 year (t = 0.849, p = 0.398).

A statistically significant difference was found between the three groups (F(2,87) = 5.022, p = 0.009). HG (28.81 UI/L ± 12.70) had higher ADA levels than both PG (20.50 UI/L ± 9.71) and RG (20.71±12.97). There was no statistically significant difference between PG and RG (U = 389.000, Z = −0.04, p = 9.998).

We divided the RG into two groups based on genetic similarity: the sibling group and another group that included the patients’ parents and children. According to ADA levels, there was no significant difference between the groups (F(25) = 0.362, t = −0.777, p = 0.445).

According to the symptom level (with CGI), there was no significant difference between the mild and severe patient groups regarding ADA levels (U = 77.500, Z = −1.163, p = 0.245). Similarly, there was no significant correlation between ADA level and any sub-dimension of the PANSS scale (respectively PANSS positive, PANSS negative, PANSS General, PANSS Total; r = 0.005, p = 0.981; r = 0.119, p = 0.537; r = 0.008; p = 0.966, r = 0.008, p = 0.966).

The patient group was composed of 25 patients using atypical antipsychotics and four patients using a combination of typical and antipsychotics. No patients were using only typical antipsychotics. While patients receiving only atypical AP were compared to those receiving typical and atypcial AP combination, there was no significant difference in the ADA level between the two groups (U = 49.000, Z = −0.063, p = 0.950). Six patients were using clozapine. In our analysis comparing patients using clozapine and those not using it, no significant difference in terms of ADA levels (U = 53.500, Z = −0.835, p = 0.404). There was no significant correlation between ADA and the duration of the disorder (r = 0.102, p = 0.597). There was no significant correlation between ADA and age (r = −0.082, p = 0.443).

### Binary Logistic Regression Analysis

It was initially determined whether the serum ADA levels were a suitable parameter for risk estimation since the group variable was the only significant variable in our study. Because all of the patients and their families had a genetic predisposition to schizophrenia and had significantly lower ADA levels than the control group, they were combined into a single group. The patient and relative groups were classified as high-risk, and healthy controls were classified as low-risk groups. After that, the predictive value of the schizophrenia risk model for serum ADA Level was determined using a binary logistic regression analysis. In this study, PG and RG were determined as high genetic risk groups, and HCG had relatively low genetic risk (HCG).

Hosmer Lemeshow goodness-of-fit results are examined (χ^2^(8) = 11.220, p = 0.190). The analysis showed that the established model is statistically significant (χ^2^(1) = 9.636, p = 0.002). Serum ADA level was found to be significant for the high-risk group (Wald (1) = 8551, Exp (B) = 1.059, p = 0.003). After that, using the Exp (B) coefficient, the relationship size was calculated with the formula ((1-Exp(B))* 100. The decrease in the serum ADA level increases the risk of being in the high-risk group by 5.9% (% 95 [CI] =1.019–0.101).

As a result of regression analysis, 46 (82.1%) of 56 high-risk participants (sensitivity) and 24 (29.4%) of 34 low-risk participants (specificity) in the model made using the data of ADA level as independent variable correctly classified and showed a total accuracy of 62.2% in classification (Table 2).

**Table 2:** Classification table

Observed		Predicted		Percent Correct	
		**Genetical risk level**			
		High risk	Low risk		
**Genetical risk level**	High risk	46	10	82.1	Sensitivity
	Low risk	24	10	29.4	Specificity
**Overall percentage**				62.2	Accuracy

## Discussion

Adenosine has been reported to be essential to brain immune responses, neuromodulation, and central nervous system development (Cunha 2001, 2016). Despite conflicting results, studies indicate that adenosine metabolism hypofunction may play a role in the pathogenesis of schizophrenia (Shen et al. 2012). This study aimed to examine the possible correlation between high-risk psychosis and ADA levels, which have been linked to schizophrenia in previous studies.

The general consensus in the literature is that ADA enzyme levels are higher in schizophrenia patients than in healthy populations (Brunstein et al. 2007; Dutra et al. 2010; Ghaleiha et al. 2011; O’Donovan et al. 2018). Besides, it was stated that there might be a possible relationship between the enzyme level and the severity of auditory hallucinations (Sasidharan et al. 2017). The results of our study, which demonstrate reduced levels of ADA enzyme in individuals with schizophrenia, contrast with those reported in previous studies. Additionally, we did not find any relationship between positive symptoms and ADA levels. We acknowledge that our findings may present a discrepancy and require further clarification. However, there are also various studies that reveal similar findings to ours. Specifically, it was reported that cognitive and behavioral neuropsychiatric symptoms are more common in patients with severe combined immunodeficiency characterized by ADA deficiency (Rogers et al. 2001). ADA is relevant to enzymatic and neurochemical effects and immunologic effects (Franco et al. 2007). Although ADA deficiency is more indicative of immunodeficiency, it was proposed that ADA deficiency might be associated with diseases of many non-immunological organ systems (Rogers et al. 2001; Whitmore and Gaspar 2016). Although our results contradict adenosine hypofunction hypotheses and related studies, it is possible to say that the results of the study caused us to turn our attention to the neuroimmunological hypothesis, considering the results showed in severe combined immunodeficiency.

In the literature it has been stated that antipsychotic drugs also cause differences in ADA enzyme levels (Ghaleiha et al. 2011; Whitmore and Gaspar 2016). An increase in serum ADA levels has been reported during treatment with typical antipsychotics (APs) and/or clozapine (Brunstein et al. 2007). In a study evaluating ADA activity in zebrafish brain membranes following treatment with haloperidol, olanzapine, and sulpiride, no significant changes in ADA activity were observed after olanzapine or sulpiride treatment, whereas a reduction in ADA activity was detected with haloperidol treatment (Seibt et al. 2015). In our study, 25 patients were using only atypical antipsychotics, and 4 patients were using a combination of typical and atypical antipsychotics. There were no patients receiving only typical antipsychotics. Additionally, only 6 patients used clozapine. We found no significant difference in ADA levels between patients receiving atypical-typical antipsychotic combinations and those receiving only atypical antipsychotics. Similarly, no significant difference in ADA levels was observed between individuals treated with clozapine and those who were not. Overall, our findings are insufficient to establish a relationship between antipsychotic use and ADA levels. The use of different tissues in previous studies may have affected the results (O’Donovan et al. 2018; Seibt et al. 2015). Future research involving carefully designed studies in patients undergoing monotherapy is needed to further elucidate the relationship between specific antipsychotics and ADA levels.

Our findings indicate that the serum ADA levels were significantly different between individuals with relatively high genetic risk (the PG and the RG) and those lower genetic risk (the HG). Specifically, the serum ADA levels were significantly higher in the HG compared to both the PG and RG, while no significant difference was observed between the PG and RG. When examining additional factors that could influence peripheral ADA levels, including gender, smoking history, clinical features, and sociodemographic characteristics, no significant associations were identified. Our study revealed that the group variable emerged as the most influential factor in differentiating ADA levels. Furthermore, confirming comparable results in patients and family members indicates a link with the neurodevelopmental features associated with schizophrenia. In the Brassilian cohort of schizophrenia patients study, which examined schizophrenia patients, low ADA enzyme activity was associated with a low-activity adenosine deaminase allelic variant. On the other hand, it was proposed that this polymorphism is infrequent (Dutra et al. 2010). The presence of different allelic variants may be responsible for the different results. In other words, obtaining different results in different populations may be related to genetic polymorphisms.

Finding that measuring serum ADA levels can distinguish schizophrenia patients and patient relatives from healthy controls is particularly significant as ADA represents an objective biochemical marker, minimizing researcher-related biases, and offers a simple, practical, and potentially valuable tool for establishing psychosis susceptibility. If possible, we assert that serum ADA measurement could be a relatively inexpensive and accessible method to assist clinicians in estimating schizophrenia risk. We performed a binary logistic regression analysis to evaluate the predictive capability of serum ADA levels. The results indicated that serum ADA measurement could distinguish individuals in the high-risk group (patients and their relatives) from those in the low-risk group (healthy controls). Importantly, to our knowledge, this study is the first to assess the utility of serum ADA levels in establishing psychosis risk, highlighting its potential significance in early intervention strategies. Although the model’s sensitivity was 82%, the overall accuracy and specificity were limited. Considering there was only a single parameter in our model, we propose that addition of other goal-oriented (target-specific) variables could substantially enhance its predictive power. Reaching this finding based on a single biomarker is particularly noteworthy given the multifactorial etiology of schizophrenia.

### Limitations

The limitation of our study is that a drug-free period was not provided for the patients to exclude the antipsychotics’ effect because it has been stated in the studies that it is not clear whether the alteration in ADA level is due to the disease or drugs. Although controlling this important confounder is difficult, additional studies should be carried out on drug-free patients. However, in these studies—which are different from ours—enzyme levels were not measured in first-degree relatives of schizophrenia patients who were not only genetically predisposed but medication-free. The results of our study are authentic and valuable in this sense. It can be suggested that a trait with an endophenotypic origin characterized by lower ADA levels might have a genetic basis. Another limitation of the study is that only one parameter could be investigated to evaluate adenosine metabolism. Future studies needed to elucidate the relations between ADA enzyme levels, receptor profiles, and the genetic features of ADA enzyme.

## Conclusion

The study’s findings underscore the significance of adenosine metabolism in the development of schizophrenia. Evaluation with serum ADA level is highly sensitive and can guide the clinician in distinguishing the low-risk group from the high-risk group in terms of schizophrenia. Our study is valuable because of its contribution to the identification of low- and high-risk groups of schizophrenia and its emphasis on preventive interventions. More research is necessary to comprehend the connections between inflammatory pathways and schizophrenia in order to identify potential factors and targets for treatment.

## Data Availability

No datasets were generated or analysed during the current study.
